# Pulmonary Placental Transmogrification in a Patient With Suspected Congenital Lobar Emphysema: A Case Report

**DOI:** 10.7759/cureus.116680

**Published:** 2026-09-21

**Authors:** Pedro Gomez, Jose F Rojas, Alejandro Cardona Palacio, Ariel A Arteta, Gustavo A Gomez

**Affiliations:** 1 Medicine, Universidad EIA, Medellín, COL; 2 Pathology, Universidad de Antioquia, Medellín, COL; 3 Pathology, Hospital Pablo Tobón Uribe, Universidad EIA, Medellín, COL; 4 Pathology, Universidad EIA, Medellín, COL; 5 Pathology, Hospital Universitario de San Vicente Fundación, Medellín, COL

**Keywords:** bullous emphysema, case report, cystic lung disease, immunohistochemistry, pulmonary placental transmogrification

## Abstract

Pulmonary placental transmogrification (PPT) is a rare benign pulmonary lesion characterized by papillary formations whose stromal architecture closely resembles placental chorionic villi. Of just over 60 cases reported worldwide, none originate from Colombia, and only one has been described in Latin America. We present the case of a 20-year-old male with a prior history of congenital lobar emphysema who presented in February 2024 with acute respiratory distress secondary to progressive bullous disease of the left lung. Chest computed tomography revealed extensive large bullae occupying the entire left lung, prompting a left pneumonectomy. Histopathological examination confirmed PPT, supported by an immunohistochemical panel demonstrating vimentin positivity in the stromal cells, TTF-1 positivity in the lining pneumocytes, and focal CD10 stromal positivity. The postoperative course was complicated by massive hemorrhage necessitating emergency re-thoracotomy, followed by refractory respiratory failure and cardiorespiratory arrest, resulting in the patient’s death. This case represents the first report of PPT from Colombia and documents a fatal perioperative outcome not previously described in the PPT literature, highlighting the potentially life-threatening surgical risks associated with pneumonectomy in the setting of extensive bullous disease and underscoring the need for heightened clinical awareness and meticulous perioperative planning.

## Introduction

Pulmonary placental transmogrification (PPT) is a rare entity with few reports in the literature, mostly isolated cases or small patient series [1,2]. A systematic search of PubMed/MEDLINE and Embase databases using the terms “placental transmogrification” and “pulmonary placental transmogrification” identified just over 60 reported cases, none from Colombia and only one from Latin America [2,3]. The condition predominantly affects men, with a reported male-to-female ratio of approximately 4:1, and most cases are diagnosed in the third to fifth decades of life [1,2]. The majority of reported cases originate from North America, Europe, and Asia, with a notable underrepresentation of Latin American populations in the literature [3,4]. Therefore, it remains largely unknown among local pathologists, pulmonologists, and thoracic surgeons.

PPT is defined as a benign pulmonary lesion characterized by the replacement of lung parenchyma by papillary formations whose stromal architecture closely resembles placental chorionic villi, composed of fibroconnective cores containing clear stromal cells and thin-walled vascular spaces, lined by a layer of reactive pneumocytes [1,5]. Although the entity was originally described as a histological variant of giant bullous emphysema [1], its exact nature remains debated, with some authors also considering a hamartomatous component given its frequent co-occurrence with pulmonary fibrochondromatous hamartoma [2,6]. Regarding etiopathogenesis, two main hypotheses have been proposed: the first considers PPT an acquired secondary phenomenon resulting from progressive emphysematous remodeling, in which repeated cycles of air trapping and parenchymal distortion lead to metaplastic papillary reorganization of the alveolar architecture [1,7]; the second postulates a hamartomatous or dysontogenetic origin, supported by the frequent co-occurrence of PPT with fibrochondromatous hamartoma [2,6]. PPT is not considered a hereditary condition, and no familial cases have been reported to date [1]. No consistent genetic or chromosomal abnormalities have been identified in association with this entity [1].

The disease exhibits a broad spectrum of symptoms and lacks pathognomonic imaging findings [1,8]. Its clinical differential diagnoses include cystic lung diseases [8,9], primary pulmonary carcinomas [2], and pulmonary mesenchymal tumors, including fibrochondromatous hamartoma [5,6]. A definitive diagnosis requires histological examination and familiarity with the entity’s features [1]. Immunohistochemistry may be necessary to rule out differential diagnoses and avoid misdiagnosis leading to inappropriate treatment [2,5].

We report this case for two principal reasons. First, it represents the first documented case of PPT from Colombia and only the second from Latin America [3], contributing to the geographical characterization of this entity in an underrepresented region. Second, unlike the majority of previously published cases in which surgical resection was followed by an uneventful recovery [1,10], our patient experienced a fatal perioperative course following pneumonectomy, constituting an outcome not previously described in the PPT literature. These aspects, together with the diagnostic challenges inherent to this entity, justify its documentation and make this case particularly relevant for pulmonologists, pathologists, and thoracic surgeons in our region.

## Case presentation

In February 2024, a 20-year-old male presented to the emergency department with respiratory distress secondary to an exacerbation of his underlying disease, initially suspected to be congenital lobar emphysema. He required supplemental oxygen, with partial improvement, leading to hospitalization. He denied other symptoms and had no relevant comorbidities beyond his known pulmonary condition. His first encounter with Pulmonology was in 2022 due to dyspnea from spontaneous pneumothorax. He had no other medical history, medications, allergies, or exposure to tobacco, drugs, chemicals, or irritants. His surgical history included thoracotomy for spontaneous pneumothorax two years prior; no histopathological or cytological examination of pulmonary tissue had been performed at that time. The patient’s respiratory complaints had been present for more than one month prior to the current presentation, although the exact duration could not be precisely determined from the available clinical information. No significant family history was reported.

Physical examination revealed normal vital signs and oxygen saturation of 94% on 28% FiO₂. Decreased breath sounds were noted over the left hemithorax. The right hand showed congenital ankylosis of the second finger; the left hand had syndactyly of the third and fourth fingers. No genetic workup was performed in this patient. No previously described association between PPT and constitutional chromosomal or genetic abnormalities has been reported in the literature [1]. The remainder of the physical examination was unremarkable.

A chest CT scan (Figure 1) revealed large bullae occupying the entire left lung, causing mediastinal shift and contralateral lung compression, with thin internal septa and nodular densities consistent with adipose tissue. These features, although not pathognomonic, warranted consideration of PPT within the differential diagnosis of cystic lung disease in this young patient; the combination of thin-walled bullae with internal fibrous septa and intralesional adipose densities extends beyond the typical appearance of congenital lobar emphysema or uncomplicated bullous disease and may serve as an imaging cue toward PPT [8,9].

**Figure 1:**
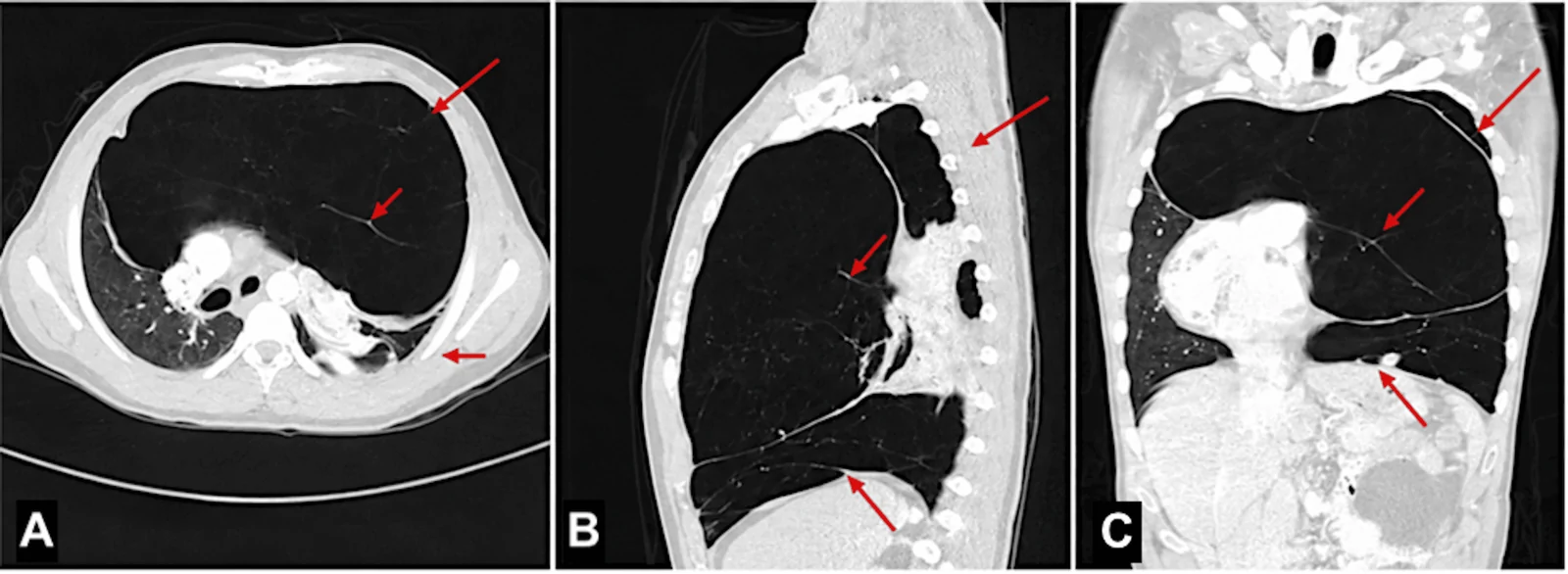
Computed tomography of the chest: (A) axial, (B) sagittal, and (C) coronal planes. Large bullae occupying the left lung, causing mediastinal shift and compression of the contralateral lung. Thin internal septa and nodular densities consistent with adipose tissue are also observed.

Initial diagnostic considerations included congenital lobar emphysema and congenital pulmonary airway malformation (CPAM) - both occurring in young patients and associated with bullous emphysema patterns [8,9]. Given the severity of left lung involvement with hyperinflation causing contralateral collapse and progressive respiratory failure, a left pneumonectomy was performed.

Histopathology received a left pneumonectomy specimen measuring 18 × 12 × 6 cm with a lobulated, smooth, hemorrhagic surface. The cut surface showed multiple cystic cavities of varying size, some hemorrhagic and others with vesicular formations. Microscopy (Figure 2) showed papillary structures lined with respiratory epithelium and fibroconnective cores. Some structures contained proteinaceous material, whereas others showed proliferation of mature adipose tissue associated with fibrous connective tissue and fibroblasts without atypia. Areas of emphysematous dilation were noted. Other lung structures were preserved. Immunohistochemical analysis demonstrated stromal positivity for vimentin in the papillary structures, TTF-1 positivity in the pneumocytes lining these structures, and focal stromal positivity for CD10 (Figure 3). The final histopathological diagnosis was PPT, with no evidence of malignancy in the examined specimen.

**Figure 2:**
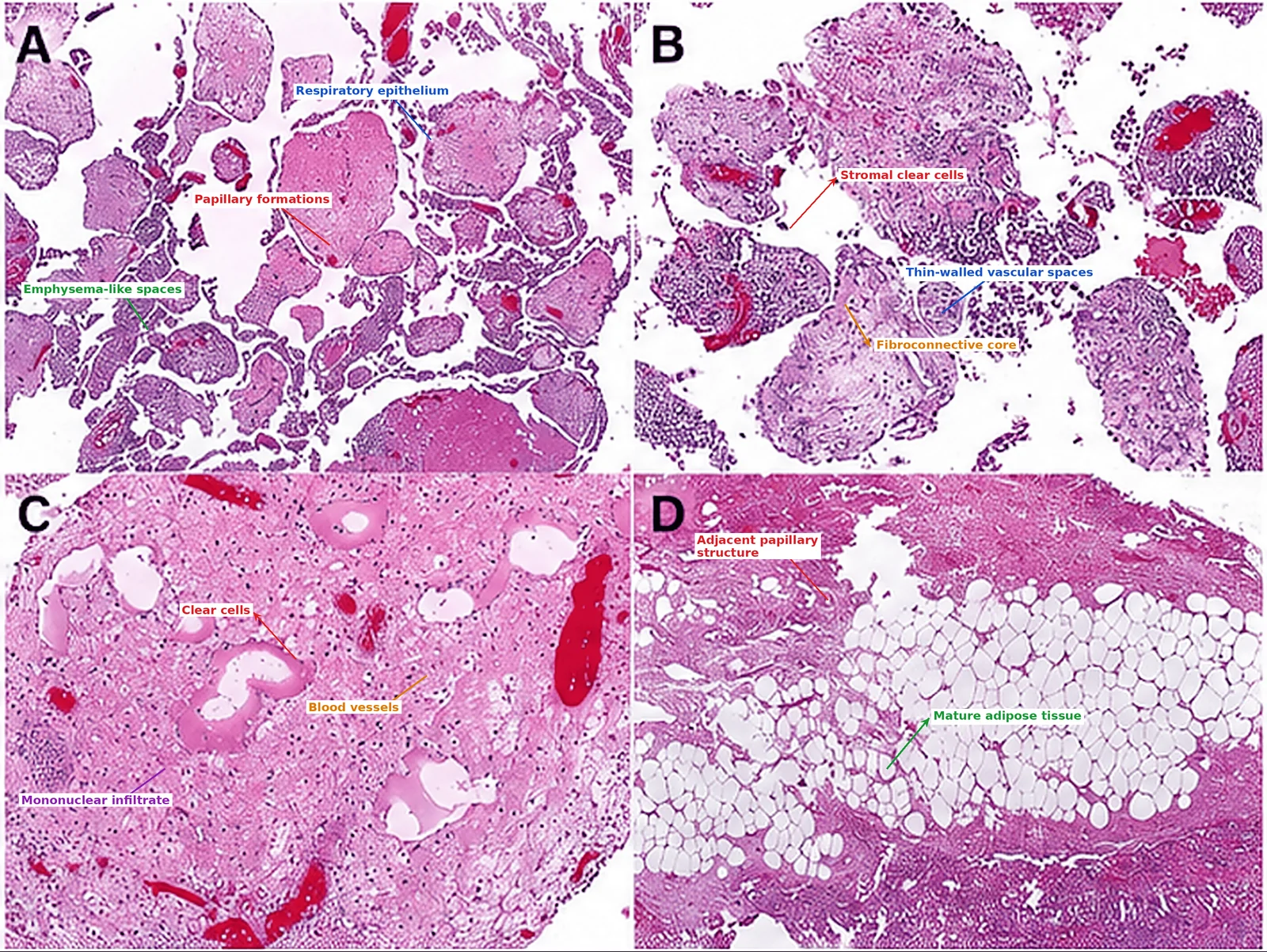
Histopathological features of pulmonary placental transmogrification (hematoxylin and eosin stain, H&E). (A) Low-power view (40×, H&E) showing multiple papillary formations lined by respiratory epithelium with fibroconnective cores, closely resembling placental chorionic villi, interspersed with emphysema-like dilated spaces. (B) Intermediate-power view (100×, H&E) highlighting the presence of stromal clear cells and thin-walled vascular spaces within the cores of the papillary structures. (C) High-power view (400×, H&E) demonstrating fibrous stroma with clear cells, blood vessels, and a sparse mononuclear inflammatory infiltrate. (D) Low-power view (40×, H&E) showing extensive proliferation of mature adipose tissue within the interstitium adjacent to the papillary structures.

**Figure 3:**
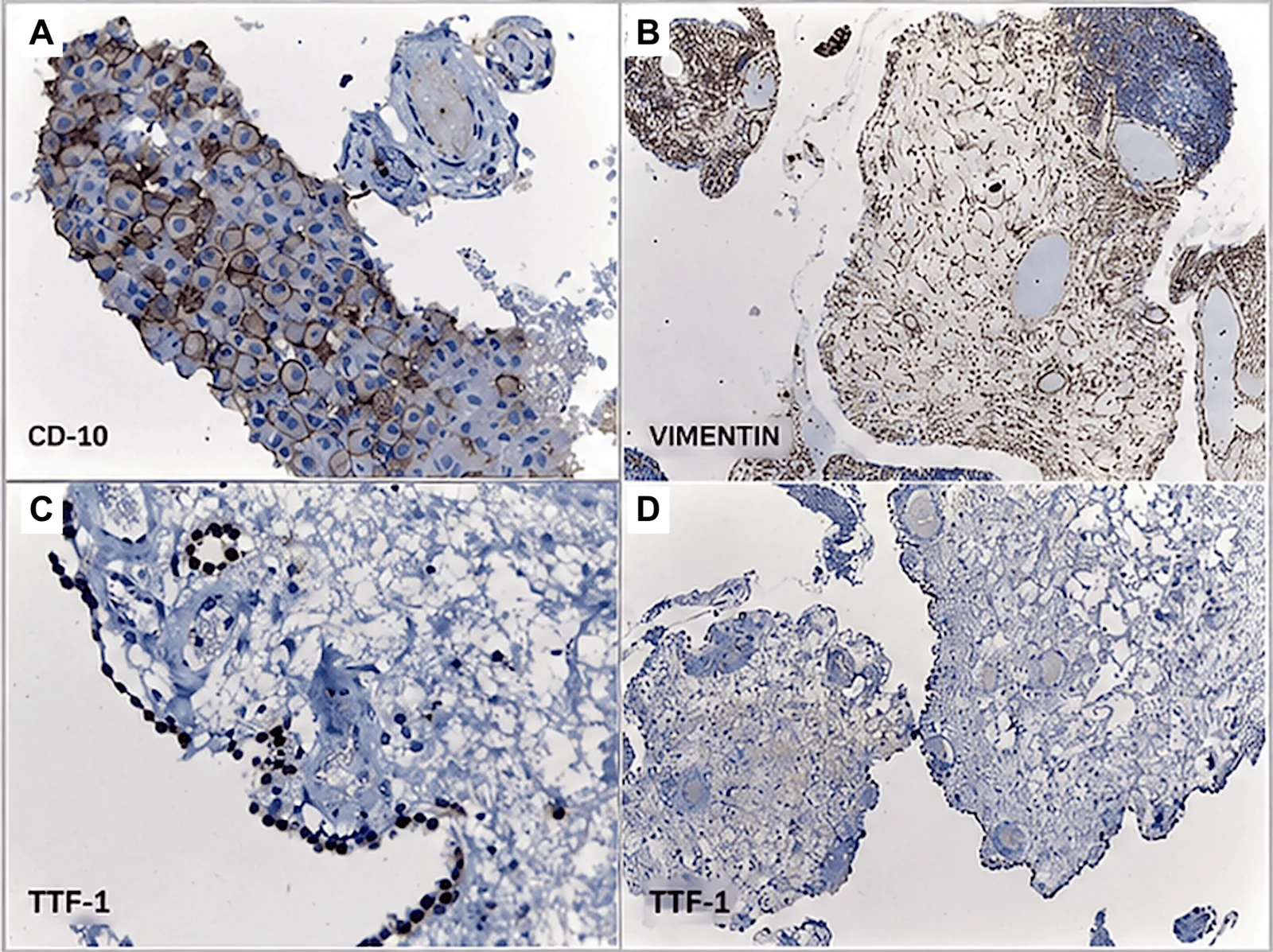
Immunohistochemical panel of the papillary structures. (A) CD10 immunostain demonstrating focal cytoplasmic and membranous positivity in the stromal cells of the papillary formations. (B) Vimentin immunostain showing diffuse strong positivity throughout the stromal component of the papillary structures, confirming mesenchymal differentiation. (C) TTF-1 immunostain (high-power) with nuclear positivity restricted to the pneumocytes lining the papillary formations, with negative stromal cells. (D) TTF-1 immunostain (low-power) demonstrating the distribution of TTF-1-positive pneumocytes across multiple papillary structures, highlighting their respiratory epithelial lining.

During the immediate postoperative period, the patient initially reported substantial improvement in his respiratory symptoms and remained hemodynamically stable for the first 24 hours. Subsequently, he developed hypotension, tachypnea, diaphoresis, and altered consciousness. Output through the pleural drainage tube was noted to be frankly hemorrhagic, with 700 mL of blood collected, prompting an emergency re-thoracotomy. Intraoperatively, approximately 2,500 mL of hematic content was evacuated; active bleeding from an intercostal muscle flap was identified, hemostasis was secured, and hemodynamic stability was restored.

Twenty-four hours following the second surgical intervention, the patient developed progressive deterioration of ventilatory mechanics with hypoxemic respiratory failure and right-sided pneumothorax. A second pleural drainage tube was placed, yielding approximately 1,200 mL of serohematic material. Despite these measures, the patient’s clinical condition continued to deteriorate. He experienced a cardiorespiratory arrest prior to a planned third surgical intervention. Emergency thoracotomy with direct cardiac massage was performed at the bedside; however, spontaneous circulation was not restored after 30 minutes of advanced cardiopulmonary resuscitation, and death was declared.

## Discussion

PPT is a rare entity that remains poorly recognized worldwide and in our region [1,2]. It predominantly affects men between 20 and 50 years of age [1,2]. Some authors situate PPT within the spectrum of cystic lung diseases, with clinical and imaging findings that may simulate other respiratory disorders, resulting in a broad differential diagnosis [8,9]. The definitive diagnosis relies on the recognition of characteristic histopathological findings [1].

In this patient, dyspnea and CT findings (Figure 1) led to initial suspicion of congenital lobar emphysema or CPAM - both encountered in young patients and associated with bullous emphysema patterns [8,9]. Some authors propose PPT as part of the bullous emphysema spectrum, though the limited number of reported cases hinders definitive clarification of this relationship [1,7]. These diagnoses were ruled out based on the histopathological findings. Beyond the pulmonary findings, this patient presented with syndactyly of the left hand and congenital ankylosis of the second finger of the right hand. However, there are currently no reports describing an association between PPT and these types of anatomical abnormalities or genetic disorders [2].

Another entity in the differential diagnosis was pulmonary hamartoma, considered due to the disproportionate mature adipose tissue proliferation observed on imaging. However, the absence of a well-defined solid or endobronchial mass, the presence of extensive cystic remodeling across an entire lung lobe, and the clinical context of a young patient with prior spontaneous pneumothorax were inconsistent with hamartoma, which effectively excluded this diagnosis [6,11].

Comparison with previously reported cases reveals both similarities and important differences. Consistent with the existing literature, our patient was a young male - the demographic most commonly affected - presenting with dyspnea and extensive bullous lung disease [1,2]. The CT findings of large thin-walled bullae with internal septa and intralesional adipose densities were concordant with the radiological patterns described by Shin et al. and Kim et al. [8,9]. Histopathologically, the papillary formations lined by respiratory epithelium with fibroconnective cores, along with the immunohistochemical profile (vimentin-positive stroma, TTF-1-positive pneumocytes, and focal CD10 positivity), are consistent with the characterization established by Hamza et al. and Cavazza et al. [2,5]. However, two aspects distinguish this case from the majority of published reports. First, whereas most cases have been managed with lobectomy or bullectomy with uneventful postoperative courses [1,10], our patient required pneumonectomy due to the extent of unilateral disease and experienced a fatal perioperative course secondary to massive hemorrhage followed by refractory respiratory failure - an outcome not previously described in the PPT literature. This underscores the significant surgical risk associated with total lung resection in the context of extensive parenchymal remodeling and pleural adhesions that may accompany longstanding bullous disease. Second, unlike approximately one-third of reported cases in which PPT co-occurs with pulmonary fibrochondromatous hamartoma [6], no hamartomatous component was identified in our specimen, supporting the notion that these two entities, while frequently associated, are not invariably linked [5].

The diagnosis of PPT is fundamentally morphological, based on identifying hallmark features including stromal proliferation with clear cells and thin-walled vascular spaces resembling chorionic villi, lined by pneumocytes that may show reactive changes (Figure 2) [1,5]. Although immunohistochemical panels including TTF-1, vimentin, D2-40, and CD10 (Figure 3) have been described [2,5], these markers primarily serve to exclude other entities in the differential diagnosis, such as pulmonary carcinomas and mesenchymal tumors, rather than to establish the diagnosis of PPT itself [2,5].

Regarding prognosis, PPT is generally considered a benign entity for which complete surgical resection is curative [1,2]. No cases of malignant transformation have been reported in the literature [2]. However, the present case demonstrates that the perioperative course is not uniformly benign, particularly when the extent of disease necessitates pneumonectomy rather than more limited resection. The fatal outcome in our patient - attributable to massive hemorrhage and subsequent cardiorespiratory failure rather than to the PPT itself - highlights the importance of thorough preoperative risk stratification, careful intraoperative hemostasis, and vigilant postoperative monitoring in patients with extensive bullous disease undergoing major pulmonary resection. Long-term follow-up data for PPT remain limited given the rarity of the condition, and reporting of additional cases is needed to better characterize outcomes across the spectrum of surgical approaches.

## Conclusions

PPT is a rare disease not previously described in our region, requiring high clinical suspicion from pathology, pulmonology, and thoracic surgery teams. Its clinical and imaging features are nonspecific and may mimic more common conditions, leading to potential diagnostic and therapeutic errors - even on histological examination. Definitive diagnosis relies on the identification of characteristic placental-like papillary structures. Immunohistochemistry serves to exclude differential diagnoses, including carcinomas and mesenchymal tumors. Critically, this case demonstrates that the perioperative course of PPT may be life-threatening when pneumonectomy is required, an outcome not previously documented in the literature. Further research is needed to assess surgical risk stratification in this entity, as well as to explore possible associations with congenital anatomical anomalies, as observed in this case.
